# Comparison of bacterial community structure and dynamics during the thermophilic composting of different types of solid wastes: anaerobic digestion residue, pig manure and chicken manure

**DOI:** 10.1111/1751-7915.12131

**Published:** 2014-06-25

**Authors:** Caihong Song, Mingxiao Li, Xuan Jia, Zimin Wei, Yue Zhao, Beidou Xi, Chaowei Zhu, Dongming Liu

**Affiliations:** 1 Life Science College, Northeast Agricultural UniversityHarbin, China; 2 Innovation Base of Groundwater and Environmental Systems Engineering, Chinese Research Academy of Environmental ScienceBeijing, 100020, China

## Abstract

This study investigated the impact of composting substrate types on the bacterial community structure and dynamics during composting processes. To this end, pig manure (PM), chicken manure (CM), a mixture of PM and CM (PM + CM), and a mixture of PM, CM and anaerobic digestion residue (ADR) (PM + CM + ADR) were selected for thermophilic composting. The bacterial community structure and dynamics during the composting process were detected and analysed by polymerase chain reaction–denaturing gradient gel electrophoresis (DGGE) coupled with a statistic analysis. The physical-chemical analyses indicated that compared to single-material composting (PM, CM), co-composting (PM + CM, PM + CM + ADR) could promote the degradation of organic matter and strengthen the ability of conserving nitrogen. A DGGE profile and statistical analysis demonstrated that co-composting, especially PM + CM + ADR, could improve the bacterial community structure and functional diversity, even in the thermophilic stage. Therefore, co-composting could weaken the screening effect of high temperature on bacterial communities. Dominant sequencing analyses indicated a dramatic shift in the dominant bacterial communities from single-material composting to co-composting. Notably, compared with PM, PM + CM increased the quantity of xylan-degrading bacteria and reduced the quantity of human pathogens.

## Introduction

Thermophilic composting (TC) is a biochemical process used to treat organic solid wastes and produce organic fertilizer (Haruta *et al*., 2005). However, the waste may not always be suitable for composting. Kumar and colleagues (2010) reported that a high moisture content in food waste or low nitrogen content of green waste may result in long treatment times or low degradation efficiencies. Co-composting is acknowledged to provide some advantages over single-material composting, such as compensating for the carbon/nitrogen (C/N) ratio of the initial mixture, providing additional biodegradable organic matter and active biomass, and improving compost quality. Zhang and colleagues (2008) reported that co-composting is a desired choice in the disposal of agricultural waste and the production of useful compost material in farms. Bustamante and colleagues (2008) studied the feasibility of co-composting the solid wastes generated by the winery and distillery industry and animal manures, concluding that it is an efficient method to treat these two types of wastes.

Co-composting processes are usually used to dispose of livestock manure. The amount of manure produced in China has dramatically increased in recent years because of the rapid development of chicken farms (He *et al*., 2012) and the pig industry (Song *et al*., 2012). Manure treatment and disposal are a critical issue for China; the inappropriate disposal of manure may result in environmental problems, such as groundwater and surface water pollution via the leaching of nitrates and other pollutants in the manure (Gao *et al*., 2010). Two of the most common substrates in the TC process, pig manure (PM) and chicken manure (CM), have been extensively used (Lau and Wu, 1987; Luo *et al*., 2008; He *et al*., 2012). However, the physical-chemical characteristics of PM and CM substrates constitute certain disadvantages for single-material composting. Likewise, single anaerobic digestion residue (ADR) is also not suitable for composting because organic matter is not easily biodegraded.

The anaerobic digestion of organic wastes creates a nutrient-rich residue that can be used as a fertilizer for agricultural soil (Fujino *et al*., 2005). Nevertheless, organic pollutants, such as pesticides, veterinary antibiotics and hormones, have been detected in both fresh and anaerobically digested waste (Nyberg *et al*., 2004). In addition, ADR is the final remnant of the original waste placed into anaerobic digesters and cannot be utilized by microbes in the anaerobic degradation process (Arthurson, 2009). It contains organic matter that is not easily biodegraded, which might lead to an insufficient composting temperature to fulfil the hygienic requirements for organic waste. Ho and colleagues (2013) revealed that the TC treatment might eliminate the negative effects of organic pollutants. Thus, co-composting is a better approach for solving the previously mentioned problems: ADR is mixed with easily degradable wastes (PM and CM) to meet the composting requirements for the C/N ratio and organic matter. Finally, the co-composting process could also eliminate harmful effects of the organic pollutants in the ADR on the soil quality.

The composting microflora is responsible for the recycling of these organic solid wastes, and their activity affects compost quality. The study of the functional diversity of microbial populations in compost is important for understanding the composting process (Poulsen *et al*., 2008). Moreover, knowledge of the microbial structure and functions (involved in the main nutrient cycles) in mature composts is important to predict its potential impact on soil fertility (Vivas *et al*., 2009). Maeda and colleagues (2010) investigated the bacterial communities in the core, bottom, top, middle-surface and lower-surface full-scale passively aerated cattle manure compost. Steger and colleagues (2007) studied the effects of different temperature regimes on the succession of actinobacteria populations during composting. Vivas and colleagues (2009) assessed the impact of composting and vermicomposting on the bacterial community size and structure, and the microbial functional diversity of an olive-mill waste. Although many researchers have investigated the structural and functional diversities of microbial communities during the composting process (Maeda *et al*., 2010), few studies have examined the characteristics of bacterial communities during the ADR and livestock manure co-composting process, and the impact of composting substrate types on the succession of bacterial populations.

In this study, the bacterial community structure for different types of composting was obtained by polymerase chain reaction (PCR)-denaturing gradient gel electrophoresis (DGGE). During the composting process, functional bacteria that correspond to specific organic matter were distinguished according to their DGGE profile. The characteristics of bacterial communities during the composting of different types of solid wastes and the impact of composting substrate types on the bacterial community structure and dynamics during the composting process were revealed.

## Results and discussion

### Physical-chemical analyses of composting experiment

**Figure 1:**
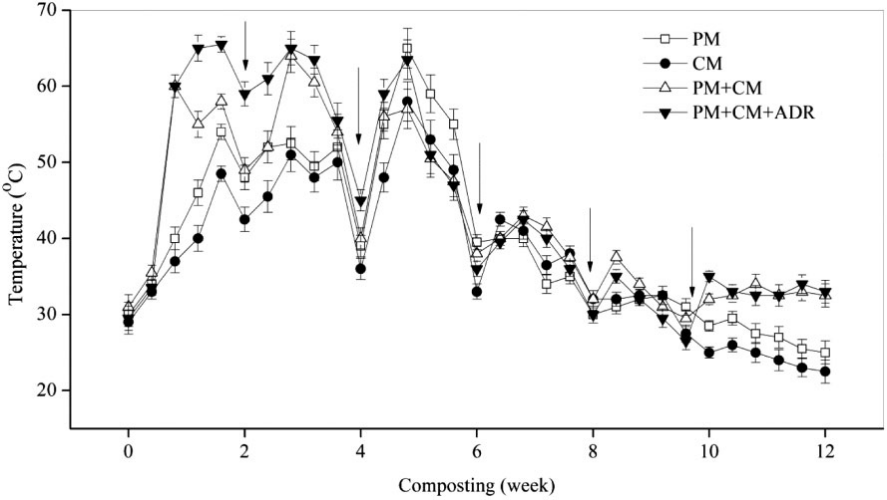
Temperature dynamics of PM, CM, the mixture of PM and CM (PM + CM), and the mixture of PM, CM and ADR (PM + CM + ADR). Arrows indicate each time of turning over. Error bars: mean ± standard deviation of two replicates.

The temperature dynamics of each composting treatment are shown in Fig. 1. A similar trend was observed for the four trials over the experimental period. The peak temperature in CM was only 58°C; the high temperature stage (above 55°C) only persisted for 67.2 h. The peak temperatures for PM, PM + CM and PM + CM + ADR were 65, 64 and 65.5°C, respectively, and the high temperature stage (above 55°C) persisted for 201.6, 443.5 and 618.2 h (the data not shown) respectively. All treatment groups except for CM met the hygienic requirements for organic waste (USEPA, 1994) [when the high temperature stage (above 55°C) lasted 3–5 days, the relevant compost product could be deemed harmless]. This finding may be due to the C/N ratio in CM (9.11 ± 0.86), which was lower than that in others (Table 1) and was not in the appropriate range of C/N ratio for microbial growth. A previous study reported that high microbial activity could lead to a rapid increase in the temperature and a period of elevated temperatures (Steger *et al*., 2007). The temperature value in the present study approximately obeyed the following order during the entire composting process: PM + CM + ADR > PM + CM > PM > CM, which indicated that the microbial activity was higher and organic matter was degraded more actively in co-composting than in single-material composting.

**Table 1:** Chemical component profiles of compost piles

	VS (%TS)	NO_3_^−^(mg/kg TS)	NH_4_^+^(mg/kgTS)	TN (g/kgTS)	C/N ratio
PM					
Initial	80.64 (6.07)^a^	19.11 (1.54)^d^	635.60 (28.14)^b^	17.87 (1.24)^d^	25.06 (1.28)^a^
Final	65.47 (4.48)^bc^	1026.20 (72.97)^b^	122.23 (9.11)^c^	22.75 (1.85)^bcd^	19.48 (1.16)^b^
CM					
Initial	45.76 (3.53)^de^	32.40 (2.28)^d^	1006.89 (72.86)^a^	24.40 (1.68)^abc^	9.11 (0.86)^d^
Final	39.61 (2.78)^e^	277.89 (19.20)^c^	138.57 (10.38)^c^	21.93 (1.73)^bcd^	8.56 (0.78)^d^
PM + CM					
Initial	72.54 (5.33)^ab^	23.39 (2.29)^d^	711.58 (34.08)^b^	20.09 (1.64)^cd^	21.50 (1.85)^ab^
Final	56.39 (4.07)^cd^	1566.85 (115.18)^a^	86.93 (5.75)^c^	27.52 (2.12)^ab^	14.24 (1.29)^c^
PM + CM + ADR					
Initial	60.84 (4.39)^bc^	57.67 (4.34)^d^	715.34 (39.27)^b^	19.64 (1.40)^cd^	22.45 (1.67)^ab^
Final	44.57 (3.30)^de^	1724.35 (127.25)^a^	31.58 (2.10)^c^	28.51 (2.00)^a^	13.57 (1.30)^c^

Values followed by different letters (a, b, c, d and e) are statistically significantly different (*P* < 0.05).

**Figure 2:**
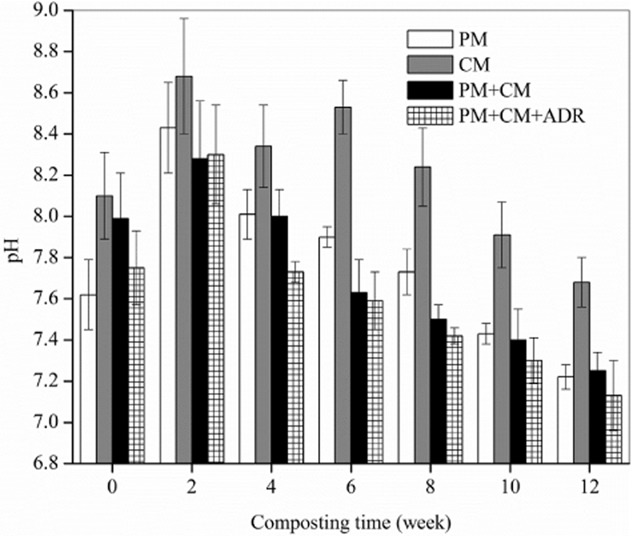
The pH dynamics of PM, CM, the mixture of PM and CM (PM + CM), and the mixture of PM, CM and ADR (PM + CM + ADR). Error bars: mean ± standard deviation of two replicates.

The variations of pH throughout the process are shown in Fig. 2. The pH increased at first and then decreased sharply. The increased pH was most likely the result of ammonia release due to ammonification (Bustamante *et al*., 2008). The subsequent decrease in pH may be attributed to the accumulation of organic acids (Li *et al*., 2013). The pH has frequently been used as an index for evaluating compost maturity and quality, and tends to be alkaline in mature compost. In this study, the pH values in PM, CM, PM + CM and PM + CM + ADR were 7.22, 7.68, 7.25 and 7.13, respectively, at the end of the composting process. All trials could reach the alkaline standard of pH in mature compost. The phenomenon was common and desirable. The pH of PM and CM, especially CM, was markedly higher than that of other trials (PM + CM and PM + CM + ADR) during the entire composting process.

Other chemical parameters are shown in Table 2. The volatile solids, NH_4_^+^-N and the C/N ratio decreased, and the NO_3_^−^-N content increased after composting for all four groups. Maeda and colleagues (2010) studied the changes in these chemical parameters during a passively aerated cattle manure composting process and obtained the same results obtained from this study. However, the increased (decreased) levels in the previously mentioned chemical parameters were higher in PM + CM and PM + CM + ADR than in PM and CM, which also supported the finding that microbial activity was higher and organic matter degraded more actively in co-composting than in single-material composting. The total nitrogen (TN) in PM, PM + CM and PM + CM + ADR increased by 27.82%, 35.57% and 44.17%, respectively, because of the concentration of dry matter in compost piles caused by the microbial mineralisation and evaporation of water. However, the TN in CM decreased by 10.61%. Silva *et al*. (2014) reported that a low initial C/N ratio may favour N losses, which may be responsible for the decrease of TN in CM at the end of composting. Alternatively, the result obtained for the TN also implied that co-composting could conserve nitrogen (Bernal *et al*., 2009).

**Table 2:** Combination ratio of the substrates fed into the reactors

Experiment	Combination of the substrates (%w/w)		
	PM	CM	ADR
PM	100	0	0
CM	0	100	0
PM + CM	75	25	0
PM + CM + ADR	50	25	25

### Bacterial community structure and dynamics

**Figure 3:**
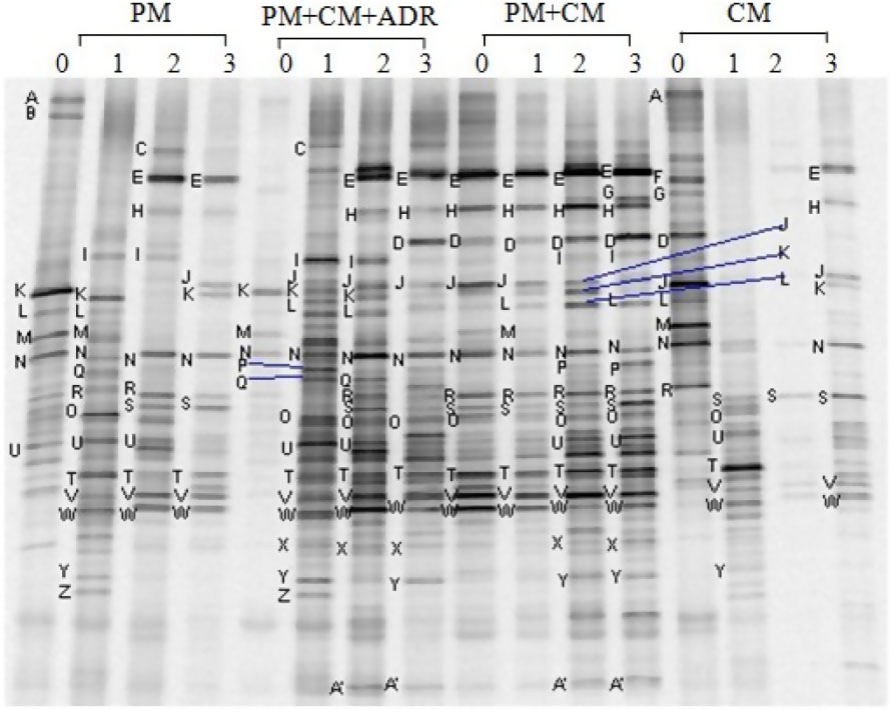
DGGE profile of 16S rDNA gene fragments amplified with the primer set R534-P3.The numbers above each lane indicate the sampling months. Band patterns of PM, CM, PM + CM and PM + CM + ADR are presented. The enclosed bands and names indicate the clone names described in Table 2.

**Figure 4:**
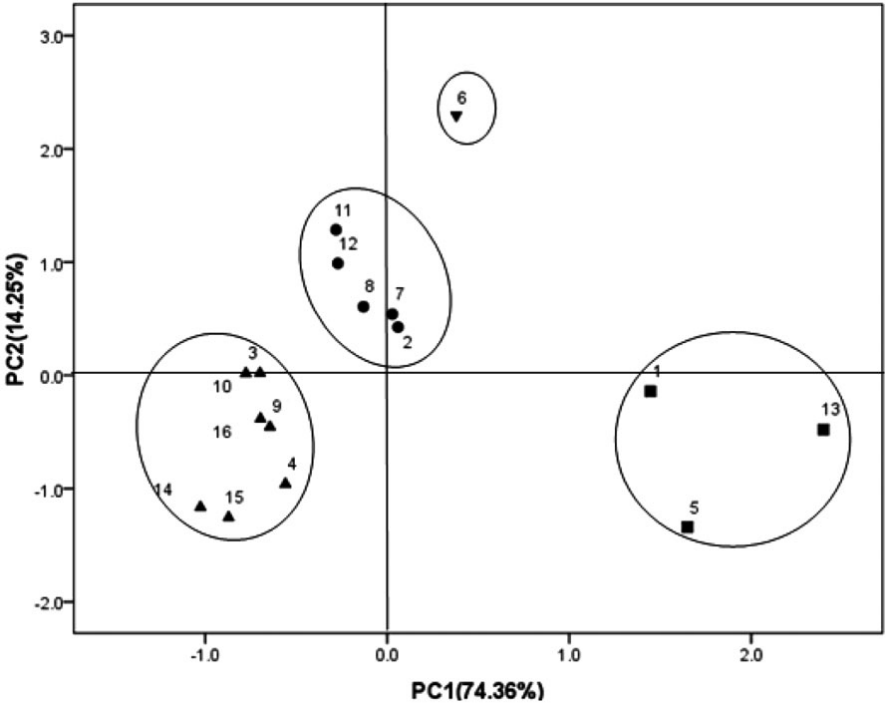
PCA of the 16S rDNA gene band patterns of PM, CM, PM + CM and PM + CM + ADR. Numbers beside the symbols indicate the sample names: 1, PM 0; 2, PM 1; 3, PM 2; 4, PM 3; 5, PM + CM + ADR 0; 6, PM + CM + ADR 1; 7, PM + CM + ADR 2; 8, PM + CM + ADR 3; 9, PM + CM 0; 10, PM + CM 1; 11, PM + CM 2; 12, PM + CM 3; 13, CM 0; 14, CM 1; 15, CM 2; 16, CM 3. The numbers after the name of composting treatment indicate the sampling months.

The DGGE band patterns of 16S rDNA gene fragments and the results of principal component analysis (PCA) are shown in Figs 3 and 4 respectively. The DGGE profiles showed a significant difference in the four composting treatments. The quantity of bands in lanes PM + CM and PM + CM + ADR clearly exceeded that in lanes PM and CM. The intensities of bands in the PM + CM and PM + CM + ADR profile were also stronger than those in lanes PM and CM. The results revealed that the co-composting process could improve the bacterial diversity. Generally, each type of raw material is inhabited by its intrinsic microbial community, which differs from that of other raw materials (Poulsen *et al*., 2008). Thus, a multiple-material composting system could introduce more bacterial species than single-material composting to the composting process. Moreover, the C/N ratio and biochemical structure of the substrate in the co-composting system were superior to those of single-material composting for bacterial growth. The PCA of the DGGE profile showed that the band patterns derived from the initial samples (1, 5, 13) approached each other in all cases except for PM + CM, which denoted that the bacterial communities for different starting substrates did not visibly differ. A temperature of 50°C was usually considered as the demarcation of the thermophilic or mesophilic phases in the composting process (Xiao *et al*., 2009). The band patterns derived from the co-composting (PM + CM, PM + CM + ADR) after the high temperature period (7, 8, 11 and 12) grouped together. The band patterns derived from single-material composting after the high temperature period (3, 4, 15 and 16) were clearly separated from those of the other samples. In addition, the band pattern derived from PM + CM + ADR at the high temperature stage (6) was remarkably different from those of the other samples.

### Diversity and similarity of bacterial communities

To better understand the differences in the bacterial communities of the four treatments, the structural diversity of the bacterial communities was also examined by the Shannon (*H’*) and Simpson (*D*) diversity index. In addition, the quantity of bands (*S*) for DGGE profiles is listed (Table 3).

**Table 3:** Shannon index of general diversity (*H’*), Simpson index of dominance (*D*) and number of bands (*S*) for DGGE profiles from PM, CM, PM + CM and PM + CM + ADR. The numbers in the first column of the table indicate the sampling months

	*H’*	*D*	*S*
PM 0	2.6075	0.0886	16
PM 1	2.1044	0.1316	9
PM 2	2.6142	0.0825	16
PM 3	2.3337	0.102	11
CM 0	2.2877	0.1105	11
CM 1	1.9018	0.1804	8
CM 2	1.5764	0.2139	5
CM 3	2.6251	0.0813	16
PM + CM 0	2.7633	0.0703	18
PM + CM 1	2.6378	0.0865	17
PM + CM 2	3.0327	0.0539	24
PM + CM 3	2.9338	0.0582	21
PM + CM + ADR0	1.7858	0.1952	7
PM + CM + ADR1	2.9688	0.0549	21
PM + CM + ADR2	2.969	0.0548	21
PM + CM + ADR3	2.8983	0.0617	21

The numbers in the first column of the table indicate the sampling months.

The Shannon–Weaver diversity index, *H’*, represents the species abundance and distribution uniformity (Dilly *et al*., 2004). The Shannon–Weaver diversity index, *H’*, and microbial diversity positively correlate. *H’* decreased at first during the high temperature stage and then increased in the PM, CM and PM + CM groups. The temperature increased significantly during the high temperature stage (Fig. 1), which could be conducive to some, but not all, initially present bacteria. Karadag and colleagues (2013) concluded that the temperature gradient significantly affects the microbial population. Steger and colleagues (2007) have observed that the species and quantity of microorganisms in a composting system during the high temperature period markedly decreased due to the screening effect of high temperatures. Shukla and colleagues (2009) also found reduced microbial diversity at high temperatures during the high temperature composting phase. Notably, *H’* increased during the high temperature stage in the PM + CM + ADR group, which was inconsistent with the earlier studies and indicated that the presence of ADR could improve the diversity of bacterial communities in a co-composting system even during the high temperature period. The environmental conditions may have been more suitable for bacterial growth in a multiple-material composting system, which weakened the screening effect of high temperatures on bacterial communities. *H’* in CM decreased significantly and did not increase until the last mature stage. The combined effects of high temperatures and the degradation of nitrogen-containing organic matter resulted in the strong volatilisation of ammonia during the high temperature stage. The volatilization of ammonia could persist for long periods until the maturation stage. Because the volatilization of ammonia could be toxic to the microorganisms, the *H’* in the CM group [high nitrogen content and significant ammonia volatilization (Table 2)] exhibited the previous change in trend.

The Shannon–Weaver diversity index *H’* roughly obeyed the following order during the entire fermentation process: PM + CM + ADR > PM + CM > PM > CM, which indicated that multiple-material composting could improve the diversity of bacterial communities and that bacterial diversity positively correlated with the type of raw material used in co-composting. Marzorati and colleagues (2008) indicated that the range-weighted richness (*Rr*) reflected the microbial diversity and carrying capacity of the system. In this study, the *Rr* for almost all lanes, PM + CM + ADR and PM + CM, was higher than 30 (a high *Rr* level), while that of the majority lanes PM and CM were lower than 30 (a medium or low *Rr* level). The phenomenon also indicated that the microbial diversity was higher for the co-composting system than for single-material composting. The results of *Rr* agreed with those of *H’*.

The Simpson index, *D*, negatively correlated with the microbial diversity. In this study, the Simpson index, *D*, and the Shannon–Weaver diversity index, *H’*, exhibited opposite trends, which revealed that the results of *D* were consistent with those of *H’*. The quantity of bands (*S*) for the DGGE profiles also demonstrated a richness of microbial communities. In this study, the results of *S* agreed with those of *H’*.

**Figure 5:**
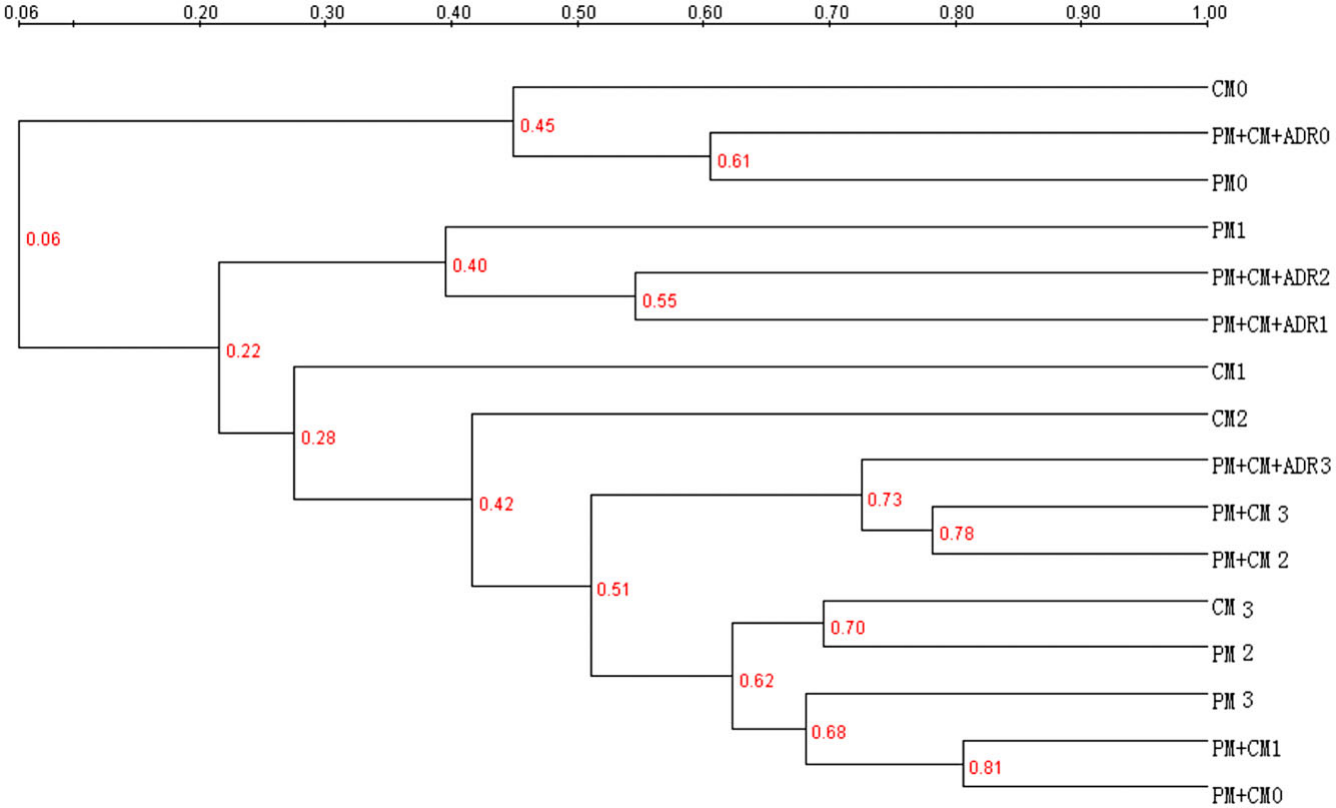
Similarity cluster analyses based on Complete Linkage algorithm for DGGE profiles for bacteria present in PM, CM, PM + CM and PM + CM + ADR. The numbers (0, 1, 2 and 3) beside composting treatment indicate the sampling months.

To compare the DGGE profiles of bacteria present in PM, CM, PM + CM and PM + CM + ADR, a dendrogram describing pattern similarities was created (Fig. 5). The dendrogram showed that PM 0, CM 0 and PM + CM + ADR 0 clustered together with a homology coefficient of 45% (PCA also showed similar result). Notably, the dendrogram showed that the samples named ‘1’ clustered closer and that the samples named ‘2’ and ‘3’ roughly clustered together with a homology coefficient of 42%. The ‘1’, ‘2’ and ‘3’ samples represented the samples in the thermophilic stage, the cooling stage and the mature stage of composting respectively. All of the earlier results indicated that the bacterial communities varied according to the composting stage, except for the small difference of bacterial communities between the cooling and the mature stages. The temperature was an important selective factor for the development of actinobacteria populations in composts (Steger *et al*., 2007). This study obtained similar findings on the effect of temperature on bacterial dynamics during the composting process. However, the PM + CM and PM + CM + ADR samples were not always in line with the earlier rule. Notably, the dendrogram showed 55% homology between the sample named ‘1’ and the sample named ‘2’ in PM + CM + ADR, while 81% homology was observed between the sample named ‘0’ and the sample named ‘1’ in PM + CM. These findings also implied that the screening effect of temperature on bacterial communities was weaker during the co-composting process than during single-material composting.

### Analysis of 16S rDNA sequences

The bands in the gel were excised to assess their signal intensities and obtain all of the information of bands in the gel. The sequences of a total of 24 DNA fragments were successfully determined (because less DNA was recovered, the bands of D, K and U were not successfully sequenced). A similarity value higher than 97% allows the best match found to be associated to a genus name (Altschul *et al*., 1997). Notably, the similarity values of bands J and Q were 95% and 93%, respectively, which indicated that band J and Q may be two new species. The sequences consisted of 21, 23, 21 and 19 dominant bands excised from the DGGE analyses of PM, PM + CM + ADR, PM + CM and CM respectively. The retrieved sequences could be classified as three main types of bacterial phyla: *Firmicutes*, *Bacteroidetes* and *Proteobacteria* (Table 4). Nissilä and colleagues (2012) studied the hydrogenic and methanogenic fermentation of a mixture of birch and conifer pulps and also found that the main bacterial phyla were *Firmicutes*, *Bacteroidetes* and *Proteobacteria*.

**Table 4:** Sequence analysis of bands excised from DGGE gels derived from bacterial 16S rDNA extracted from compost samples

Band position	Accession No.	Closest sequence available in database(%similarity)	Similarity (%)
A	HQ731452.1	*Acinetobacter sp*. BN-HKY5	100
B	EU845582.1	Uncultured bacterium clone 1103200832100	100
C	EU551120.1	Uncultured *Bacteroidetes* bacterium clone B9	100
E	DQ141183.1	*Ruminofilibacter xylanolyticum* strain S1	100
F	JQ269284.1	Bacterium WHC3-10	100
G	JN834841.1	Uncultured *Bacteroidetes* bacterium clone 181T2TEAT	99
H	FN436048.1	Uncultured bacterium clone HAW-R60-B-727d-F	99
I	HQ433472.2	*Pseudomonas sp*. A84(2010)	100
J	AB436739.1	*Desulfonosporus sp*. AAN04	95
L	JQ268616.1	Uncultured anaerobic bacterium clone XA3	100
M	JF915345.1	*Acinetobacter junii* strain NW123	100
N	HQ731452.1	*Acinetobacter sp*. BN-HKY5	98
O	EF629964.1	Uncultured *alpha proteobacterium* clone 6mML2F12	100
P	FJ675561.1	Uncultured bacterium clone LL141-8K15	100
Q	CU918603.1	Uncultured *Firmicutes* bacterium clone QEEA1AB12	93
R	JX426171.1	Uncultured bacterium clone bac4_6-1-2D8	97
S	FN667411.1	Uncultured compost bacterium clone PS2677	100
T	AM491470.1	*Psychrobacter sp*. Nj-69 16S rRNA gene, strain Nj-69	100
V	EF414143.1	Uncultured *alpha proteobacterium* clone MPWIC_A11	100
W	HQ224821.1	Uncultured bacterium clone ABRB33	100
X	DQ141183.1	*Ruminofilibacter xylanolyticum* strain S1	100
Y	JN368260.1	Uncultured bacterium clone LSW-L1-126	100
Z	JN834841.1	Uncultured *Bacteroidetes* bacterium clone 181T2TEAT	100
A’	EU551120.1	Uncultured *Bacteroidete*s bacterium clone B908061601-ZSS_YX_Z8_EU_2_46	100

In the present study, bands E, H, J, K, L, M, N, O, R, S, T, U, V, W and Y were detected in all trials. Five bands of *Proteobacteria* corresponding to the genus *Acinetobacter* (M and N), *Psychrobacter* (T) and uncultured bacterium (O and V) were recovered. Band E belonged to *Ruminofilibacter* in *Bacteroidetes*. In *Firmicutes*, band J was closely related to the genus *Desulfonosporus*. The remaining clones (H, L, R, S, W and Y) were classified as unclassified bacteria. In addition to the previously mentioned common bands, the dominant sequences from PM also included the bands named A, B, C, I, Q and Z. Bands A and I were ascribed to *Acinetobacter* and *Pseudomonas* in *Proteobacteria* respectively. Bands C and Z were affiliated with the uncultured bacterium in *Bacteroidetes*. Band Q belonged to the uncultured bacterium in *Firmicutes*. Band B was classified as an unclassified bacterium. In addition to the common bands, the dominant sequences from PM + CM + ADR included the bands named C, D, I, P, Q, X, Z and A’. Bands X and A’ belonged to *Ruminofilibacter* and uncultured bacterium in *Bacteroidetes* respectively. Band P was classified as an unclassified bacterium. In addition to the common bands, the dominant sequences from PM + CM also included bands named D, G, I, P, X and A’. Band G were ascribed to the uncultured bacterium in *Bacteroidetes*. In addition to the common bands, the dominant sequences from CM included bands named A, D, F and G. Band F was classified as an unclassified bacterium.

Dominant sequencing analyses indicated a shift in the dominant bacterial communities from PM to PM + CM and from PM + CM to PM + CM + ADR. Compared with PM, bands A, B, C and Q disappeared, and bands G, X and A’ appeared in PM + CM. Band A was affiliated with *Acinetobacter* in *Proteobacteria*. *Acinetobacter* contains a number of human pathogens (Bergogne-Bérézin and Towner, 1996). Band X belonged to *Ruminofilibacter xylanolyticum*, which is a rumen bacterium and related to the degradation of xylan (Nissilä *et al*., 2012). The composting of the mixture of PM and CM increased the quantity of DGGE bands, which were ascribed to the *Bacteroidetes* family and reduced the quantity of DGGE bands affiliated with the *Proteobacteria* and *Firmicutes* families. Notably, PM + CM increased the quantity of xylan-degrading bacteria and reduced the quantity of human pathogens. Likewise, compared with PM + CM, band G disappeared, and bands C and Q appeared in PM + CM + ADR, which indicated that the addition of ADR to the PM and CM compost mixture increased the quantity of DGGE bands affiliated with the *Firmicutes* family. Similarly, dominant sequencing analyses indicated a shift in the dominant bacterial communities from CM to PM + CM and from PM + CM to PM + CM + ADR. Compared with CM, bands A and F disappeared, and bands I, P, X, Z and A’ appeared in PM + CM, which denoted that the addition of CM to PM increased the quantity of DGGE bands in the compost mixture classified as *Bacteroidetes*.

The earlier shift in the dominant bacterial communities could be due to the ‘priming effect’ and antagonistic interaction (which could stimulate the generation of new bacterial species and kill some bacterial species) between the microflora in one organic solid waste and that in another one or two composting materials. In addition, the nutrient composition and physicochemical environment are a function of the composting substrate (shown in the previously mentioned physical-chemical analyses), which may also result in changes in the bacterial communities for different composting trials (Poulsen *et al*., 2008).

## Conclusions

In this study, co-composting could degrade organic matter more actively and conserve more nitrogen than could single-material composting. The co-composting process, especially PM + CM + ADR, could improve the bacterial community structure and functional diversity during the composting process, even during the high temperature period. The environmental conditions may have been more suitable for bacterial growth in the co-composting system, which weakened the screening effect of high temperature on the bacterial communities. The dominant bacterial communities shifted from single-material composting to co-composting. Notably, compared with PM, PM + CM increased the quantity of xylan-degrading bacteria and reduced the quantity of human pathogens. The shift most likely occurred due to the ‘priming effect’ and antagonistic interaction between the microflora, and the changes in the nutrient composition and physicochemical environment.

## Experimental procedures

### Preparation of composting substrates

PM and CM were collected from a pig farm and a chicken farm, respectively, near Laoting County (in Hebei Province) during May 2012. After scraping off the hog-hair and feathers, the samples were immediately transported to the laboratory and stored in a refrigerator at approximately 4°C until they were used as substrates for the composting experiments (less than 15 days). The ADR was collected from a farm in the Shunyi District, Beijing, during May 2012. It was pretreated by screening out the stones and then ground. The total solids (TSs) contents in PM, CM and ADR were 28.77% ± 3.59%, 26.58% ± 3.24% and 17.55 ± 2.66% respectively. The volatile solids (VSs) contents in PM, CM and ADR were 80.64% ± 6.07%, 45.76% ± 3.53% and 35.70% ± 1.34% respectively.

### Composting setup and sampling

PM, CM, PM + CM and PM + CM + ADR were selected as composting substrates. Each substrate was placed in duplicate 30 l reactors for composting from the 15th of May until the 15th of August. During the composting process, the moisture level was maintained at approximately 60%.

The combination ratios of the substrates fed into the reactors and the characteristics of the substrates are shown in Tables 1 and 2 respectively. The oxygen was supplemented via ventilation (0.5 l kg^−1^ h^−1^). The pH and temperature were measured every 12 h. The compost piles were turned over artificially once every 2 weeks. Compost samples (200 g wet weight) were removed once a month. Each sample was divided into two parts. One part (100 g wet weight) was stored at −20°C to be used for the molecular experiment, and the remainder (100 g wet weight) was air-dried to measure the chemical parameters.

### Analytical methods

#### Physical and chemical analysis

The temperatures of the compost piles were measured with a Thermo Recorder RTW-30S (Espec). The pH of the aqueous extracts (solid-to-water ratio of 1:10, w/v) of the compost samples was measured with a SevenEasy pH metre S20K (METTLER TOLEDO). This dilution ratio was defined based on the water absorbance of the tested compost samples. The inorganic N (NO_3_^－^-N and NH_4_^+^-N) in the previously mentioned aqueous extracts was measured using an ICS 5000 ion chromatograph (DIONEX).

The TS, VS, TN and C/N ratio were measured according to Maeda and colleagues (2010).

#### DNA extraction and purification

The composting samples were pretreated as follows:

1. Three grams of the sample was suspended in a 50 ml centrifuge tube with 10 ml of PBS buffer (pH 8.0), 10 ml of removing humic substance buffer solution and 500 mg of glass beads. The sample was then vortexed to ensure even mixing and centrifuged at 1000 r.p.m. for 10 min.
2. The supernatant in step (i) was transferred into another 50 ml centrifuge tube. Ten millilitres of PBS buffer (pH 8.0) and 10 ml of removing humic substance buffer solution were added to the precipitate in step (i), and the sample then vortexed to ensure even mixing and centrifuged at 1000 r.p.m. for 10 min.
3. The supernatant in step (ii) was mixed with that in step (i) and then centrifuged at 1000 r.p.m. for 10 min. The supernatant was transferred into a 50 ml centrifuge tube and then centrifuged at 1000 r.p.m. for 10 min. This step was repeated three times to remove impurities.
4. The supernatant obtained at the end of step (iii) was centrifuged at 13 000 r.p.m. for 5 min, and the supernatant was discarded.

The total DNA was extracted from the precipitate of step (iv) with the E.Z.N.A.™ Soil DNA kit (Omega Biotek, Inc.) in accordance with the manufacturer’s instructions. The concentration and quality of the DNA samples were assessed using a NanoDrop ND-1000 Spectrophotometer (NanoDrop Technologies).

#### PCR-DGGE analysis

PCR was conducted with the 16S rDNA universal bacterial primers R534 and GC-F341 [Sangon Biotech (Shanghai) Co.] as described by Nubel and colleagues (1996). The PCR program was conducted mainly according to the procedure of Vivas and colleagues (2009) with some revisions. The program was initiated by a hot start of 5 min at 94°C; after 5 min of initial denaturation at 94°C, a touchdown thermal profile protocol was used, and the annealing temperature was decreased by 1°C per cycle from 68 to 58°C; 20 additional cycles were then performed at 58°C. The amplification was performed with 30 s of denaturation at 94°C, 30 s of primer annealing and 1 min of primer extension at 72°C, followed by a final primer extension at 72°C for 10 min. The total reaction mixture of the first PCR consisted of the following ingredients for a final volume of 50 μl: 2 μl of extracted DNA, 1 μl of 10 μM primer R534, 1 μl of 10 μM primer P3, Premix Taq 25 μl [TaKaRa Biotechnology (Dalian) Co., Ltd.] and sterile Milli-Q water to a final volume of 50 μl. The second amplification was performed using 2 μl of the products of the first reaction as a template. The second PCR was performed in accordance with the protocol of Vivas and colleagues (2009).

DGGE analyses were carried out using 20 μl of the latter PCR product loaded into polyacrylamide (8%) gels with gradients of 35–60% of denaturants (urea/formamide). A Gene Mutation Detection System (Bio-Rad Laboratories, Inc.) was run at 80 V for 16 h at 60°C to separate the fragments. The gels were silver stained with the Bio-Rad Silver Stain (Bio-Rad Laboratories, Inc.) according to the standard DNA-staining protocol and photographed under UV light using a Gel Imaging System (Bio-Rad Laboratories, Inc.).

The sequences were analysed, and the excised bands were identified according to Vivas and colleagues (2009). The results are shown in Table 4.

#### Statistical analysis

The statistical significance of differences between all replicated samples was determined via an analysis of variance following Duncan’s multiple range comparison tests using the SPSS 17.0 (SPSS International) software package. The differences in means were compared at *P* < 0.05. The relative intensity data of the DNA bands from the bacterial communities were used to carry out the following analyses and calculations: a PCA of the 16S rDNA gene band patterns was performed using the earlier software. The Shannon–Weaver diversity index, *H’*, (Shannon and Weaver, 1963) and Simpson index of dominance, *D*, (Simpson, 1949) were calculated from the quantity and relative intensities of bands present in each lane according to Vivas and colleagues (2009). The digital image was analysed, and the similarity cluster analyses based on Complete Linkage algorithm were generated by Quantity one v4.62 (Bio-Rad) software to express the relatedness of bacterial communities as similarity clusters.
