# Comparison of bacterial community structure and dynamics during the thermophilic composting of different types of solid wastes: anaerobic digestion residue, pig manure and chicken manure

**DOI:** 10.1111/1751-7915.12310

**Published:** 2015-08-11

**Authors:** Caihong Song, Mingxiao Li, Xuan Jia, Zimin Wei, Yue Zhao, Beidou Xi, Chaowei Zhu, Dongming Liu

**Affiliations:** 1 Life Science College, Northeast Agricultural UniversityHarbin, China.; 2 Innovation Base of Groundwater and Environmental Systems EngineeringChinese Research Academy of Environmental Science, Beijing 100020, China.

In the originally published article by Song and colleagues (2014), the affiliation for author Yue Zhao was incorrectly stated as ‘*Innovation Base of Groundwater and Environmental Systems Engineering, Chinese Research Academy of Environmental Science, Beijing 100020, China*’.

The correct affiliation should have read ‘*Life Science College, Northeast Agricultural University, Harbin, China*’*.*
